# Assessment of Cancer Patients’ Mental Health during the Coronavirus Pandemic in Serbia

**DOI:** 10.3390/ijerph20021132

**Published:** 2023-01-09

**Authors:** Tamara D. Klikovac, Nikola Petrović, Đerđi Šarić

**Affiliations:** 1 Department of Psychology, Faculty of Philosophy, University of Belgrade, 11000 Belgrade, Serbia; 2 Department of Oncology, General Hospital Radivoj Simonović, 25000 Sombor, Serbia

**Keywords:** cancer patients, mental health, coronavirus, depression, anxiety, stress

## Abstract

This study aimed to determine the impact of the COVID-19 pandemic on the mental health of cancer patients in Serbia. Data were collected between April–May 2021 using an online questionnaire, which estimated depression, anxiety, and stress in a sample of 221 cancer patients. The Depression, Anxiety, and Stress Assessment Scale (DASS-21) was used to assess the cancer patients’ affective status. The T test of independent samples and the one-way analysis of variance (ANOVA), as well as multiple linear regression analysis, have been used as well. The results showed that moderate to extremely severe depression, anxiety, and stress symptoms were present in 33.9%, 21.2%, and 26.7% of patients, respectively. Older patients and those who assessed their socioeconomic and health status as better were less anxious, depressed, and stressed. The study shows that the patients who have stated that medical help has been available significantly differ from those patients who have not had available medical help, meaning that they have shown lower scores on the scales of depression and stress. When it comes to the availability of medical help during the pandemic, statistically significant differences among patients on the scale of anxiety have not been proven. It has been determined that statistically important differences exist between patients who have needed psychological help and those who have not needed it when it comes to the results on all of the three subscales. The patients who have expressed the need for psychological help have higher levels of depression, anxiety, and stress. The patients who have not had available psychological help have higher levels of depression, anxiety, and stress when compared to the patients who have had available psychological help.

## 1. Introduction

Since the start of the Coronavirus disease 2019 (COVID-19) pandemic, over 630 million cases and over 6.5 million deaths were reported until October 20 worldwide [1]. Serbia is currently facing the seventh pandemic wave, with over 1300 new cases a day [2]. There are no precise data available regarding the number of cancer patients among new coronavirus cases or deaths in Serbia. A diagnosis of malignant disease and undergoing combination or other oncology therapy with uncertain prognosis are considered life crises by both patients and family members. Psycho-oncology research has shown that cancer patients are vulnerable and at particular risk of developing various psychological problems, ranging from expected distress, demoralization, fear, worry, anxiety, and depressive reactions to developing anxiety and depressive disorders, PTSD, mood and adjustment disorders, etc. [3,4,5,6,7,8,9,10,11,12,13].

The current pandemic poses an extraordinary threat to people suffering from malignant diseases with their associated immune dysregulation. Fears of contracting COVID include a more severe infection and side effects of its therapy, as well as concerns about the potential exacerbation of the primary disease with potentially fatal outcomes, are a threat to the mental health of cancer patients. Those fears are shared by those who have completed their therapy or those still under treatment. Patients who started or are in the process of oncology treatment during the pandemic faced additional delays in scheduling their treatments, as well as challenges in scheduling follow-up appointments, which could also cause various psychological reactions and issues [14,15,16]. Research on cancer patients’ mental health during the 2 year pandemic has shown variable results. A Multinational Cross-Sectional Study was carried out in 78 countries during the first pandemic wave, namely the lockdown period (April 2020–June 2020), with the aim of explaining how people around the world reacted to the beginning of the pandemic. A large sample of 9565 participants (264 of these were cancer patients) assessed the level of distress, anxiety, the prevalence of positive/negative emotions, and wellbeing. The results showed that cancer patients reported lower levels of distress and depression, more psychological flexibility, higher prevalence of positive affect and wellbeing, and greater self-efficacy in adhering to recommended national guidelines, regarding COVID-19 protective behaviors against Coronavirus, compared to the control group without malignancy diagnoses [17]. In contrast to the above results, other studies have shown that distress, depression, anxiety, insomnia, and various fears are the most common psychological problems cancer patients have faced during the COVID-19 pandemic [18,19,20,21,22,23,24,25,26,27,28]. Research by Swainston et al. has shown that the disruption of common, available oncology services during the COVID-19 pandemic is associated with higher levels of anxiety and depression in cancer patients [29]. In a study examining the impact of COVID-19 on quality of life and psychological wellbeing in 1051 female breast cancer patients, the authors found a decline in wellbeing was associated with reduced direct contact with doctors. Several studies have confirmed that changes in treatment and concerns about not being able to meet with doctors are critical factors affecting high levels of anxiety in cancer patients during the pandemic [19,20,30,31,32,33,34]. The general goal of this research was to look into the levels of depression, anxiety, and stress from the available sample of oncology patients. The research question that we have acted upon was whether the oncology patients will differ based on the levels of depression, anxiety, and stress when compared to the sociodemographic characteristics that we have included (age, residency, education, socioeconomic status, self-evaluation of the health status), as well as in comparison with the availability of medical help, the need for psychological help, and the availability of it during the course of pandemic.

A cancer diagnosis is always a shock. People suffering from cancer face fears, anger, sadness, distress, disappointment, despair, feelings of hopelessness and emptiness, and often anxiety and depression reactions. The complex oncological treatment represents a life crisis both for the affected person and for the members of their family [35]. Taking care of the mental health of oncology patients is as important as oncology treatment. In Serbia, psycho oncology has been slowly developing, and it has been in the process of being implemented in the health system with many obstacles [35]. The general goal of this research was to look into the levels of depression, anxiety, and stress from the available sample of oncology patients. The research question that we have acted upon was whether the oncology patients will differ based on the levels of depression, anxiety, and stress when compared to the sociodemographic characteristics that we have included (age, residency, education, socioeconomic status, self-evaluation of the health status), as well as in comparison with the availability of medical help, the need for psychological help, and the availability of it during the course of pandemic. There are very few research studies conducted on cancer patients during the global pandemic, especially in Serbia, and while the pandemic has slowed down, the significance of this study is in the observation structures of oncology patients who are active in associations and online (many of them do not use social networks). The goal was also to see how they felt at the time of the pandemic, which provides a framework for the target group towards which it would design and implement psycho-social intervention, which is not sufficiently developed in Serbia. 

## 2. Materials and Methods

### 2.1. Study Design and Participants 

The study was conducted in accordance with the approval of the Institutional Review Board of the University of Belgrade, Faculty of Philosophy, Department of Psychology (Approval number: #2021-22), which approved the study. This research is part of a more extensive study related to assessing the mental health of cancer patients in Serbia during the pandemic. Due to numerous limitations caused by the pandemic and the impossibility of carrying out research in one of the oncology centers in Serbia, we decided to collect data through social network profiles of various cancer patient associations. An anonymous online questionnaire in Serbian with informed consent, demographic data, oncological diagnosis, and COVID-19 related questions—the DASS-21 scale (21 items)—was created using the Google Docs platform. The demographic data was collected because, in previous similar studies, it proved to be important [36]. The associations that shared the questionnaire link on their social network profiles and whose members participated in the research are: National Association of Cancer Patients—NALOR; Vojvodina Association of Cancer Patients—VALOR; ‘Budimo zajedno’ Association of Women with Breast Cancer; ‘Progovori’ Citizens’ Association for the Fight Against Ovarian Cancer; Hrast Leskovac Association of Cancer Patients; Association of Melanoma Patients. Data collection took place from April 2021 until May 2021. 

Incomplete online questionnaires were excluded from the analysis. There were 233 cancer patients who completed the questionnaires. Of these, 12 questionnaires were not valid and, therefore, not processed, so the total number of cancer patients who participated in the study was 221. The informed consent has been required from the questionees by checking the option “I agree to participate in this research”. Before checking the consent, the research goals were presented, the research was presented in more detail, and we stated the data about the researchers and the ethical consent for conducting this research. We have disclosed the e-mail of the researchers in case of additional questions, suggestions, etc.

### 2.2. Measures 

The online questionnaire included a brief study description and invitation to participate. Demographic data included sex, age, employment status, residence, level of education, marital status, and socioeconomic status. Age was categorized into three groups: young adults (ages 18–35), middle-aged adults (ages 36–55), and older adults (ages 56–81). Employment status was categorized into four groups: student, employed, unemployed, and retired. Participant residence was ranked into cities (more than 100,000 inhabitants), towns (more than 10,000 inhabitants), countryside, and Belgrade as a capital (metropolis with over two million inhabitants). Education level was categorized into five groups: elementary school, high school, college, undergraduate studies, and master/PhD. Marital status was categorized as single, married, divorced, widowed, and partnership. Socioeconomic status was categorized into below average, average, and above average. These variables were considered important, as we could assume that, for example, not having finances would affect the chances of finding adequate psychological help. The same goes for age, the level of education, and the place of residence, as younger people and less educated people have less information about such services and some parts of the country have far fewer services such as these. Finally, the marital status is an important factor of social support critical for cancer patients and their struggle with the disease.

The second part of our questionnaire included questions related to health status—oncological disease status, general health issues, and COVID-19 psychological impact—such as location of oncological disease, time since diagnosis, current status of disease, self-estimated general health status, medical services availability during the pandemic, fear of contracting COVID-19, and the need for psychological support during the pandemic. The questions we used to measure the health status of the respondents were constructed by consensus of two oncologists and two psychologists, during an online webinar for patients, during the first wave of the pandemic. We thought that the questions would be discriminatory and would point out some differences. The third part of our questionnaire was used to assess the mental health status of cancer patients and included the standardized DASS-21 questionnaire. We have opted for the DASS-21 scale since it is a short instrument, it is easy to apply, it is practical for being given on an online platform, it successfully divides the symptoms of depression, anxiety, and stress, and it provides relevant data for a faster screening of mental health. The DASS-21 questionnaire consisted of 21 statements divided into three subscales: one for depression, one for anxiety, and one for stress, each containing seven statements. The participants selected how much they agreed with each statement for the past seven days. Answers for each statement were categorized on an ordinal scale and had the following values: 0 for not at all, 1 for somewhat, 2 for quite often, and 3 for always. The sum of seven scores of each subscale multiplied by two determined its final score. This questionnaire was shown to accurately measure levels of depression, anxiety, and stress during the COVID-19 outbreak in other countries, as well as during previous pandemics [37,38,39]. This questionnaire was also previously validated on a Serbian student population with reported Cronbach’s alpha values of α = 0.92 for the entire DASS-21 scale, α = 0.85 for the depression subscale, α = 0.81 for the anxiety subscale, and α = 0.84 for the stress subscale [40]. In this study, the DASS-21 scale was found to have high general reliability (α = 0.95), as were subscales for depression (α = 0.93) and stress (α = 0.91). Subscale for anxiety (α = 0.85) had slightly lower, but still desirable, reliability. The depression score was calculated by adding scores from questions 3, 5, 10, 13, 16, 17, and 21 and multiplying by 2. The subscale of depression comprises the points that evaluate the basic symptoms of depression: a low positive effect, dysphoria, helplessness, the lack of interest, inertion, and a negative attitude towards themselves as patients and towards life in general. Certain symptoms that belong to the criteria of depressive episodes towards DSM-IV (such as problems with sleep, appetite, and concentration) are excluded from the subscale of depression, since it has been shown that they represent weak signs of depression, when it comes to both clinic samples and those that are not clinic. The anxiety score was calculated by adding scores from questions 2, 4, 7, 9, 15, 19, and 20 and multiplying by 2. The subscale of anxiety includes the points that relate to the symptoms of physiological excitement (such as dry mouth, difficulties breathing, trembling), as well as a subjective feeling of anxiety effects. The stress score was calculated by adding scores from questions 1, 6, 8, 11, 12, 14, and 18 and multiplying by 2. The subscale of stress is used to assess the symptoms of general, unspecified excitement, such as a difficulty for a person to relax and calm down, irritability, everyday nervousness, agitation, and sensitivity. The final depression score was divided into five categories: low (score 0–9), mild depression (score 10–13), moderate depression (score 14–20), severe depression (score 21–27), and extremely severe depression (score 28+). The final anxiety score was similarly divided into five categories: low (score 0–6), mild anxiety (score 7–9), moderate anxiety (score 10–14), severe anxiety (score 15–19), and extremely severe anxiety (score 20–42). The final stress score was also divided into five categories: normal (score 0–10), mild stress (score 11–18), moderate stress (score 19–26), severe stress (score 27–34), and extremely severe stress (score 35–42). We adopted ranges of each subset, as recommended by the original authors (Lovibond and Lovibond) of the DASS-21 scale [41]. The same categorization was also used in other published studies [42,43,44].

### 2.3. Statistical Analysis 

Statistical analysis was performed using SPSS Statistics Software (IBM SPSS Statistics for Windows, Version 22.0, Armonk, NY, USA). The results are shown in a table. Descriptive statistics are presented (frequencies and percentages for categorical data, as well as arithmetic means and standard deviations for quantitative data). The correlation coefficients for quantitative data were applied to determine the correlation between the sociodemographic characteristics of patients and the results on the DASS-21 scale. In order to determine an impact of predictor variables on the level of depression, anxiety, and stress, a standard multiple regression has been applied (all independent variables are introduced in the model simultaneously with a view to determine the prediction power of each independent variable). The *t*-test of independent samples, multiple linear regression and the one-way analysis of variance (ANOVA) have been used as well. The Cronbach’s Alpha reliability coefficient was used to check the reliability of the instruments used.

## 3. Results

### 3.1. Demographic Characteristics of the Analyzed Sample 

The highest percentage of the sample comprised women (97.3%), and the average age was 50.42 ± 10.2 (age range from 24 to 81). Observed by categories, (63.3%) participants belonged to the category of middle-aged adults (age 36 to 55), (31.7%) were older adults (age 56 to 81), and the lowest percentage (5%) were young adults (age 18 to 35). Additionally, one-third of the participants lived in a city (32.1%), one third in a town (33.5%), while one in five participants lived in Belgrade (21.7%), and the lowest percentage in the countryside (12.7%). Half of the participants completed high school (51.1%), 27.6% had done undergraduate studies, 14.9% had a college diploma, and 3.6% had a master’s or PhD. Only 2.8% of the participants had elementary school only. As for the employment status, half of the participants were employed (49.3%), a third was retired (33.3%), (17.7%) were unemployed, while there was no student population among the participants. The majority of the participants (73.3%) assessed their socioeconomic status as average, while (14.9%) and (11.8%) assessed their socioeconomic status below and above average, respectively. Regarding marital status, the sample was dominated by married participants (65.2%) and divorced participants (18.1%), while about 5–6% were single, in a partnership, or widowed. The sample demographic characteristics are listed in Table 1.

### 3.2. Health Status of Participants

All participants (100%) were cancer patients, of which (53.4%) were currently on oncology therapy (12 participants were excluded from the final sample due to incomplete questionnaires). In terms of disease localization, the highest percentage of the sample comprised women with breast cancer (84.2%), while a significantly lower percentage of participants reported other primary cancer locations (uterus, lung, blood, and lymph malignancies, as well as others). In addition, the majority of participants (28.5%) were diagnosed 3–5 years prior, (22.6%) in the period from 1–2 years prior, (14%) were diagnosed 2–3 years prior, and (12.7%) within a year (Table 2). Approximately one-fifth of the participants from our sample, (19.5%), contracted the COVID-19 virus, while the highest percentage did not (80.5%). A total of (27.6%) of participants reported they were afraid of contracting the COVID-19 virus, (37.6%) said they were not afraid, while a third (34.8%) felt fear to the same extent as when the pandemic began. Almost half of the participants (46.6%) assessed their general health condition as good, one-third (35.7%) as average, and (11.3%) as excellent, while about 6% of them believed that their health condition was bad/very bad. Around one-third of the participants (33.5%) believed that medical services were available to them during the pandemic, while a similar percentage reported having difficulties with scheduling appointments (36.6%), and about 30% of the participants stated medical services were not available to them. When asked about the extent to which medical services were available to them, one-third of patients (34.4%) reported that the situation was the same as before the pandemic, another third (32.1%) reported that the services were less available, (29%) reported that the services were significantly less available, while for (4.5%) participants, medical services were not available at all during the pandemic. As for the need for psychological support during the pandemic, only one-quarter of patients stated they needed psychological support. Regarding psychological support availability, participants’ opinions were polarized—50% of them reported support was available, while the other half believed the opposite (Table 2).

### 3.3. DASS-21 Questionnaire 

Scores on the DASS-21 depression subscale show that slightly more than half of the participants (54.3%) had low depression, and (17.2%) had a score indicating severe and extremely severe depression. A total of (60.6%) of participants had a low score on the anxiety subscale, and (9%) participants had severe or extremely severe anxiety levels. When it comes to the stress subscale results, a low score was reported by (33.5%) participants, mild stress level by 38.5%, and severe or extremely severe by a total of (13.1%) (Table 3). 

### 3.4. Analysis of the Correlation between Participants’ Sociodemographic Variables and DASS-21 Scale Scores 

The correlation analysis results illustrate a statistically significant correlation between the patients’ ages and the anxiety subscale scores (r (219) = −0.148, *p* = 0.028) (Table 4). The resulting correlation is low and negative, meaning the older the participants, the lower their anxiety level. In addition, there is a statistically significant correlation between the participants’ ages and the stress subscale scores (r (216) = −0.182, *p* = 0.007). The resulting correlation is low and negative, meaning the older the participants, the lower their stress level. No statistically significant correlation was found between age and depression level (*p* > 0.05). No statistically significant correlations were found between the residence and education and depression, anxiety, and stress levels (*p* > 0.05). A statistically significant correlation was reported between the participants’ socioeconomic status and the depression subscale scores (r (219) = −0.192, *p* = 0.004)). The resulting correlation is low and negative, meaning that the better the participants’ socioeconomic status, the lower their depression level. In addition, there is a statistically significant correlation between the participants’ socioeconomic status and the stress subscale scores (r (216) = −0.166, *p* = 0.014)). The resulting correlation is low and negative, meaning that the higher the participants’ socioeconomic status, the lower their stress levels. No statistically significant correlation was found between socioeconomic status and the degree of anxiety (*p* > 0.05) (Table 4). 

The correlation analysis results indicate a statistically significant correlation between the participants’ self-assessments of the general health condition and the depression subscale scores (r (219) = −0.391, *p* = 0.000)). The correlation is moderately strong and negative, meaning the better self-assessed general health, the lower the depression levels of patients. In addition, there is a statistically significant correlation between the participants’ general health self-assessments and the anxiety subscale scores (r (219) = −0.287, *p* = 0.000)). Finally, there is a statistically significant correlation between the participants’ general health self-assessments and the stress subscale scores (r (216) = −0.274, *p* = 0.000)). The illustrated correlations are weak and negative, meaning the better self-assessed general health, the lower anxiety and stress levels of patients.

### 3.5. Standard Multiple Regression, t-Test of Independent Samples and the *One*-*Way* Analysis of Variance (*ANOVA*)

Out of the conditions necessary for performing a standard multiple regression, sample size (the sample is large compared to the number of predictors) and absence of multi-collinearity (strong connections among predictor variables) have been met, as it had been determined that predictor variables had not correlated highly among themselves. The final model contains five independent variables (age, place of living, education, socio-economic status, and estimates of general health condition). The condition that the level of correlation between independent and dependent variables is above 0.3 has not been met, unless it is about the estimate of general health condition. As some variables, such as the place of living, are nominal, they had to be recoded for this analysis. In Table 5, the reader can see the summary model for depression, anxiety and stress.

From Table 6, one can see that F = 8.107 and that it is statistically significant (*p* < 0.001). This indicates that the combination of predictor variables statistically significantly contributes to the prediction of the level of depression of the patients. Therefore, there is a linear connection between a group of predictors on one side and the criterion variable on the other side. The existence of this linear connection means that a certain percentage of differences among the patients, in respect of the expression of depression, can be explained based on the fact that they differ in respect of predictor variables included in the model. For anxiety, F = 4.855, and it is also statistically significant (*p* < 0.001). This indicates that the combination of predictor variables statistically significantly contributes to the prediction of the level of anxiety of the patients. Therefore, there is a linear connection between a group of predictors on one side and the criterion variable on the other side. The existence of this linear connection means that a certain percentage of differences among the patients, in respect of the expression of anxiety, can be explained based on the fact that they differ in respect of predictor variables included in the model. F = 5.590 is statistically significant (*p* < 0.001), which indicates that the combination of predictor variables statistically significantly contributes to the prediction of the level of stress of the patients (Table 6.). The existence of the linear connection between the group of predictors on one side and the criterion variable on the other side means that a certain percentage of the differences among the patients, in respect of the expression of stress, can be explained based on the fact that they differ in respect of the predictor variables included in the model.

Based on the effect of the partial contributions of the factors (β coefficients that show the size of the effect of the prediction of a dependent variable for each individual factor), one can see that predictor variables *Age* (β = −0.163, t = −2.554, *p* = 0.011), Socio-economic status (β = −0.158, t = −2.315, p = 0.022), and Estimate of general health condition (β = −0.415, t = −6.588, p < 0.001) statistically significantly predict the differences in the expression of depression of the patients when all predictor variables are included in the model together (Table 7). Based on the effect of partial contributions of the factors (β coefficients that show the size of the effect of the prediction of the dependent variable for each individual factor), one can see that predictor variables Age (β = −0.186, t = −2.793, p = 0.006) and Estimate of general health condition (β = −0.332, t = −5.033, p < 0.001) statistically significantly predict the differences in the expression of anxiety of the patients when all predictor variables are included in the model together (Table 7).

Based on the effect of partial contributions of the factors (β coefficients that show the size of the effect of the prediction of the dependent variable for each individual factor), one can see that predictor variables *Age* (β = −0.225, t = −3.369, *p* = 0.001), Socio-economic status (β = −0.151, t = −2.120, *p* = 0.035), and Estimate of general health condition (β = −0.314, t = −4.786, *p* < 0.001) statistically significantly predict the differences in the expression of stress of the patients when all predictor variables are included in the model together (Table 7).

Table 8 shows that there are statistically significant differences among the patients, in respect of the availability of medical assistance during the pandemic (yes, no, yes, but with difficulties at scheduling the appointments), on the Depression scale, where F (2218) = 4.305 and *p* = 0.015. The subsequent multiple comparison tests (Tukey) have determined that the patients indicating that medical assistance had been available to them statistically significantly differ from the ones to whom medical assistance had not been available in the sense that they have less expressed scores on the scale of depression. No statistically significant differences have been determined among the patients, in respect of the availability of medical assistance during the pandemic, on the scale of Anxiety, where F (2218) = 1.543 and *p* = 0.216. It has been determined that there are statistically significant differences among the patients, in respect of the availability of medical assistance during the pandemic on the *Stress* scale, where F (2218) = 5.039 and *p* = 0.007. The subsequent multiple comparison tests (Tukey) have determined that the patients indicating that medical assistance had been available to them statistically significantly differ from the ones to whom medical assistance had not been available in the sense that they have less expressed scores on the scale of stress.

The *t*-test of independent samples has determined that there are statistically significant differences between the patients with the need for psychological support and the ones without it in respect of the results on the Depression scale. Namely, the patients that have expressed the need for psychological support have a higher level of depression (M = 19.61, SD = 11.30) compared to the ones that have not done so (M = 8.02, SD = 8.61), t (77.782) = 7.013, *p* < 0.001. It has also been determined that there are statistically significant differences between two groups of patients on the Anxiety scale. Namely, the patients that have expressed the need for psychological support have a higher level of anxiety (M = 13.93, SD = 9.32) compared to the ones that have not expressed the need for psychological support (M = 7.08, SD = 6.82), t (75.951) = 5.061, *p* < 0.001. It has been determined that there are statistically significant differences between two groups of patients on the *Stress* scale. The results obtained indicate that the patients that have expressed the need for psychological support have a higher level of stress (M = 23.04, SD = 8.76) compared to the ones that have not done so (M = 12.22, SD = 8.06), t (216) = 8.464, *p* < 0.001 (Table 9). By using the *t*-test of independent samples, it has been determined that there are statistically significant differences among the patients to whom the psychological support has been available and the ones to whom it has not in respect of the results on the Depression scale. Namely, the patients to whom the psychological support has not been available have a higher level of depression (M = 13.13, SD = 11.34) compared to the ones to whom it has been available (M = 8.69, SD = 9.32), t (214.215) = −3.192, *p* = 0.002. It has also been determined that there are statistically significant differences between two groups of patients on the Anxiety scale. Namely, the patients to whom the psychological support has not been available have a higher level of anxiety (M = 10.25, SD = 8.98) compared to the ones to whom it has been available (M = 7.31, SD = 6.73), t (207.379) = −2.755, *p* = 0.006. It has been established that there are statistically significant differences between two groups of patients on the Stress scale. The results obtained indicate that the patients to whom the psychological support has not been available have a higher level of stress (M = 16.87, SD = 9.73) compared to the ones to whom it has been available (M = 12.99, SD = 8.83), t (216) = −3.072, *p* = 0.002.

## 4. Discussion

Described results showed that about one year since the official declaration of the pandemic (WHO declared a pandemic on 11 March 2020), when our study was carried out on a sample of 221 cancer patients, 33.9%, 21.2%, and 26.7% had moderate to extremely severe symptoms of depression, anxiety, and stress, respectively. However, a closer investigation found severe and extremely severe symptoms in a lower percentage of participants (9% had severe and extremely severe symptoms of anxiety, 17.2% severe to extremely severe symptoms of depression, and 13.1% severe to extremely severe symptoms of stress). Comparing to the study of mental status in the current pandemic on a sample of the general population in Serbia (28.9%, 36.9%, and 38.1% reported moderate to extremely severe symptoms of depression, anxiety, and stress, respectively) reveals that the sample of cancer patients studied in the described research has slightly more depression symptoms but fewer anxiety and stress symptoms [40]. A multinational study showed that cancer patients, compared with participants without oncological disease, had lower levels of perceived stress and depressive symptoms. The same study found that cancer patients had a more positive and less negative affect and higher levels of psychological flexibility compared to people without a cancer diagnosis, suggesting that they are more resilient to unknown and threatening situations caused by the current pandemic and have a more flexible and positive way of accepting and understanding, as well as more effective coping mechanisms, as they have already faced similar threats through their primary oncological disease [17]. In contrast to our study, the results of several other studies have shown higher levels of anxiety and stress in cancer patients, which were mainly associated with current cancer treatment, hospital admission, fear of contracting COVID-19, and symptoms worsening [24,45]. Studies on large samples of cancer patients from China also show that patients tend to have poorer mental health parameters compared to the general population [19,20,21,23]. In contrast to our results, in a Mexican study, a higher percentage of patients, 44.2%, reported symptoms of severe to very severe depression and anxiety, and patients with higher scores on the depression subscale were more likely to experience delayed treatments compared with patients without depressive symptoms [46]. Our study found that older patients were less anxious and less stressed, and patients who assessed their socioeconomic status as better had lower levels of depression and stress. Thus, age and socioeconomic status proved to be important protective factors in the sample of our participants. Older patients can be assumed to be more resilient since, before the pandemic, they were diagnosed with malignant disease and treated and, thus, gained experience in overcoming crises. Better socioeconomic status can also be considered a protective factor, as it alleviates everyday existential problems and various stressful situations. One of the findings in our patient sample is that those who assess their general health as better are less depressed, anxious, and stressed compared to those who assess their health as poor. This finding is similar to that of previous studies of protective and risk factors for developing depression in women treated for cancer, which showed that poorer perception of health is a risk factor for developing depressive symptoms [47]. The result that we have gathered was expected, and it is related to those patients having lower scores on the scales of depression and stress, as well as having available medical help during the pandemics in comparison with the patients who have not had available medical help. The finding that has surprised us the most is that, when it comes to the scale of anxiety, the differences between the patients related to the availability of medical help has not been proven. Similar results indicating that suspended or limited standard medical services during the pandemic, concerns about changes in treatment, and inability to meet with physicians cause higher levels of anxiety, stress, and depression have been obtained by other authors [29,31,32,33,34]. The expected finding of this study is that patients who felt they needed psychological help and support during the pandemic were more likely to have severe to extremely severe depression and increased stress levels, while patients who claimed they did not need psychological help had the expected level of depression and stress to a greater extent. This finding indicates that it is vital to organize easily accessible (SOS help line, online support through platforms and social media, etc.) and effective psychological support for cancer patients in all phases of treatment, even post-treatment. The assessment of distress (before the pandemic) on a sample of 40 cancer patients in Serbia, a year and a half after the end of treatment, showed that, in a significant percentage of patients, emotional problems (concern, anxiety, various fears, sadness, irritability, and tension) were more pronounced compared to other problems in the post-treatment period [6]. The importance of psychological support during the pandemic is demonstrated by the fact that those patients who received psychological support during the pandemic reported a lower degree of severe stress, while patients who did not have this type of support reported severe stress levels more frequently. An association between the availability of psychological support during the pandemic and stress was also illustrated, suggesting that patients who did not have access to psychological support during the pandemic had higher levels of severe stress. A potential weakness with our study was the large number of women completing the survey compared with males. Malignancies are often gender-specific, and some personality traits may be higher in one gender or another, so the imbalance in gender may have impacted our findings. Nonetheless, this report demonstrates the importance of psychological support. An additional weakness of this study was the unavoidable need to execute the study online due to marked restrictions in social contacts and opportunities to enroll participants. The research question has been confirmed by the results that the patients who have higher levels of depression, anxiety, and stress are those who have stated that they have had the need for the psychological help during the pandemics and who have not had available psychological help. The findings contribute to the fact that the need for a professional psychological support and the unavailability of such help reflect on the mental health of oncology patients (especially during the course of the pandemics). On the one hand, practical significance of our research is a confirmation that (un)availability of medical help and the need and (un)availability of psychological help have impacted the mental status of oncology patients during the course of the pandemic. On the other hand, these findings can point out the importance of available professional help for oncology patients, and these can enable associations for oncology patients in Serbia to support faster achievement of standards in the field of psycho-oncology in Serbia (establishing departments, centers for professional psychological, psycho-social, psychotherapeutic, and psychiatric help, and support to oncology patients). The main limitation of this study is the number of male participants compared to the number of female participants, so the results may not be applicable to both genders. One additional major limitation is that we do not have information about the depression, stress, and anxiety levels in the participants before the research was conducted.

## 5. Conclusions

Despite the difficult conditions for conducting the research during pandemics, the research goals that have been related to the assessment of mental status of oncology patients and the analysis of potential factors that are connected to higher and lower levels of depression, anxiety, and stress on the subject sample of oncology patients, pointed out the importance of available medical and psychological help, i.e., services for patients. The existence of need for psychological support for oncology patients is an important finding of this research, and it can help associations of oncology patients to try establishing services, advice agencies, SOS phone numbers for available professional psychological, and psychotherapeutic help for oncology patients in Serbia.

## Figures and Tables

**Table 1:** Sociodemographic characteristics of study participants.

Variable	Category	Total Number (*N*)	Percentage (%)
Sex	Males	6	2.7
	Females	215	97.3
Age	Young Adults (18–35)	11	5
	Middle-Aged Adults (36–55)	140	63.3
	Older Adults (56–81)	70	31.7
Employment status	Student	0	/
	Employed	109	49.3
	Unemployed	39	17.7
	Retired	73	33
Residence	City (more than 100,000)	71	32.1
	Town (more than 10,000)	74	33.5
	Countryside	28	12.7
	Belgrade	48	21.7
Education level	Elementary school	6	2.8
	High school	113	51.1
	College	33	14.9
	Undergraduate studies	61	27.6
	Master/PhD	8	3.6
Marital status	Single	12	5.4
	Married	144	65.2
	Divorced	40	18.1
	Widowed	13	5.9
	Partnership	12	5.4
Socioeconomic status	Below average	33	14.9
	Average	162	73.3
	Above average	26	11.8

**Table 2:** Health status.

Variable	Category	Total Number (*N*)	Percentage (%)
Disease localization	Breast	186	84.2
	Uterus	10	4.5
	Blood and lymph malignancies	5	2.3
	Lungs	2	0.9
	Other	18	8.1
Time of diagnosis	Less than 1 year	28	12.7
	1 to 2 years	50	22.6
	2 to 3 years	31	14
	3 to 5 years	63	28.5
	6 to 10 years	27	12.2
	More than 10 years	22	10
Current oncology therapy	Yes	118	53.4
	No	103	46.6
Getting sick with COVID-19	Yes	43	19.5
	No	178	80.5
Fear of contracting COVID-19	Yes	61	27.6
	No	83	37.6
	Same	77	34.8
General health status	Very bad	2	0.9
	Bad	12	5.5
	Average	79	35.7
	Good	103	46.6
	Excellent	25	11.3
Medical services availability during the pandemic	Yes	74	33.5
	No	66	29.9
	Yes, but with difficulty scheduling appointments	81	36.6
Medical services availability extent	Same as before the pandemic	76	34.4
	Less	71	32.1
	Significantly less	64	29
	Not available at all	10	4.5
Need for psychological support during the pandemic	Yes	56	25.3
	No	165	74.7
Psychological support availability	Yes	108	48.9
	No	113	51.1

**Table 3:** Depression, Anxiety, and Stress Scale—21 items (DASS-21) final scores.

Subscales DASS-21	Category	Total Number (*N*)	Percentage (%)
DASS-21 Depression Score M ± SD: 10.96 ± 10.61	Low Mild Moderate Severe Extremely severe	120 26 37 15 23	54.3 11.8 16.7 6.8 10.4
DASS-21 Anxiety Score M ± SD: 8.81 ± 8.08	Low Mild Moderate Severe Extremely severe	134 40 27 10 10	60.6 18.1 12.2 4.5 4.5
DASS-21 Stress Score M ± SD: 15.00 ± 9.49	Low Mild Moderate Severe Extremely severe	74 85 30 20 9	33.5 38.5 13.6 9.0 4.1

**Table 4:** Correlation between age and general health assessment with DASS-21 subscales.

Characteristics		Depression	Anxiety	Stress
Age	Pearson Correlation	−0.099	−0.148 *	−0.182 **
	Sig. (2 − tailed)	0.142	0.028	0.007
	N	221	221	218
General health status	Pearson Correlation	−0.391 **	−0.287 **	−0.274 **
	Sig. (2 − tailed)	<0.001	<0.001	<0.001
	N	221	221	221

** Correlation is significant at 0.01. * Correlation is significant at 0.05.

**Table 5:** Summary model for Depression, Anxiety and Stress.

Model	R	R²	Corrected R	Standard Error of Estimate
Depression	0.459	0.210	0.184	9.853
Anxiety	0.371	0.138	0.109	7.624
Stress	0.396	0.157	0.129	8.856

Note. R = coefficient of multiple correlation; R² = coefficient of determination; Corrected R² = corrected coefficient of determination.

**Table 6:** ANOVA Depression, Anxiety, and Stress.

Model		Sum of Squares	df	Average Square	F	*p*
Depression	Regression	5211.559	7	744.508		
	Residuals	19,561.075	213	91.836	8.107	<0.001
	Total	24,772.633	220			
Anxiety	Regression	1975.679	7	282.240		
	Residuals	12,381.715	213	58.130	4.855	<0.001
	Total	14,357.394	220			
Stress	Regression	3068.711	7	438.387	5.590	<0.001
	Residuals	16,469.289	210	78.425		
	Total	19,538.000	217			

Note. df = degrees of freedom; F = statistical factor; *p* = significance.

**Table 7:** Regression coefficients of Depression, Anxiety, and Stress.

	Model	Non-Standardized Coefficients	Standardized Coefficients		T	*p*	Tol.	VIF
		B	SE	β				
	Constant	42.191	5.275		7.998	0.000		
Depression	Age	−0.169	0.066	−0.163	−2.554	0.011	0.911	1.098
	Res.Town	2.494	2.144	0.111	1.163	0.246	0.406	2.464
	Res. City	2.928	2.166	0.129	1.352	0.178	0.406	2.462
	Res. Bgd	2.141	2.342	0.083	0.914	0.362	0.446	2.244
	Education	0.579	0.734	0.055	0.790	0.431	0.777	1.287
	Socio-economic status	−3.249	1.404	−0.158	−2.315	0.022	0.793	1.261
	Estimate of general health condition	−5.565	0.845	−0.415	−6.588	0.000	0.932	1.073
Anxiety	Constant	30.034	4.197		7.156	0.000		
	Age	−0.147	0.053	−0.186	−2.793	0.006	0.911	1.098
	Res.Town	0.685	1.706	0.040	0.401	0.689	0.406	2.464
	Res. City	0.369	1.723	0.021	0.214	0.831	0.406	2.462
	Res. Bgd	−1.397	1.863	−0.071	−0.750	0.454	0.446	2.244
	Education	−0.298	0.584	−0.037	−0.511	0.610	0.777	1.287
	Socio-economic status	−0.384	1.117	−0.025	−0.344	0.731	0.793	1.261
	Estimate of general health condition	−3.382	0.672	−0.332	−5.033	0.000	0.932	1.073
Stress	Constant	1.098	4.912		8.398	0.000		
	Age	2.464	0.062	−0.225	−3.369	0.001	0.901	1.110
	Res.Town	2.462	1.984	0.044	0.443	0.658	0.410	2.438
	Res. City	2.244	2.013	0.116	1.172	0.243	0.410	2.437
	Res. Bgd	1.287	2.164	0.006	0.061	0.951	0.447	2.236
	Education	1.261	0.681	0.090	1.253	0.212	0.778	1.286
	Socio-economic status	1.073	1.311	−0.151	−2.120	0.035	0.789	1.268
	Estimate of general health condition	1.098	0.792	−0.314	−4.786	0.000	0.934	1.071

Note. B = partial contribution; SE = standard error; β = standardized partial contribution; T = Student’s test for determining statistical significance; *p* = significance. Res. Town = Residence Town, Res. City = Residence City, Res. Bgd = Residence Belgrade.

**Table 8:** Comparison of average values (scores) on the DASS−21 scale according to the availability of medical assistance during the pandemic.

Availability of Medical Assistance during the Pandemic		Sum of Squares	df	Average Square	F	*p*	LS	df1	df2	df3	Mean
Depression	Between the groups	941.274	2	470.637			6.617	2	218	0.002	8.22
	Inside the groups	23,831.360	218	109.318	4.305	0.015					13.27
	Total	24,772.633	220								11.58
Anxiety	Between the groups	200.445	2	100.222			2.878	2	218	0.058	7.51
	Inside the groups	14,156.949	218	64.940	1.543	0.216					9.79
	Total	14,357.394	220								9.21
Stress	Between the groups	874.820	2	437.410			5.290	2	215	0.006	12.47
	Inside the groups	18,663.180	215	86.805	5.039	0.007					17.48
	Total	19,538.000	217	470.637							15.30

Note. df = degrees of freedom; F = statistical factor; *p* = statistical significance; LS = Leven’s statistics.

**Table 9:** Comparison between two groups of respondents (there is and there isn’t need for psychological support) and the ones with the available psychological support and the ones without it in respect of average values (scores) on the DASS-21 scale.

	Need for Psychological Support	N	M	SD	t	*p*	Leven’s F	Test	Sig.
Depression	Yes	56	19.61	11.3	7.013	0.001 **			
	No	165	8.02	8.61			5.604		0.019
Anxiety	Yes	56	13.93	9.32	5.061	0.001 **			
	No	165	7.08	6.82			8.682		0.004
Stress	Yes	56	23.04	8.76	8.464	0.001 **			
	No	162	12.22	8.06			2.057		0.153
	Availability of Psychological Support								
Depression	Yes	108	8.69	9.32	−3.192	0.002 **			
	No	113	13.13	11.34			5.604		0.019
Anxiety	Yes	108	7.31	6.73	−2.755	0.006 **			
	No	113	10.25	8.98			8.682		0.004
Stress	Yes	105	12.99	8.83	−3.072	0.002 **			
	No	113	16.87	9.73			2.057		0.153

Note. N = number of respondents; M = arithmetical means; SD = standard deviation, t = statistical factor; *p* = statistical significance, ** *p* < 0.01.; Levene’s Test for Equality of Variances.

## Data Availability

The data that support the findings of this study are available from the corresponding author (T.K.) upon reasonable request.

## References

[1] Worldometer COVID-19 Coronavirus Pandemic 2020. https://www.worldometers.info/coronavirus/.

[2] The Ministry of Health of the Republic of Serbia, The Institute of Public Health “Dr. Milan Jovanović Batut”. COVID-19 Statistical Data. https://covid19.rs/homepage-english/.

[3] Cook S.A., Salmon P., Hayes G., Byrne A., Fisher P.L. (2018). Predictors of emotional distress a year or more after diagnosis of cancer: A systematic review of the literature. Psycho-Oncology.

[4] Klikovac T., Đurđević A. (2010). Psychological aspects of the cancer patients’ education: Thoughts, feelings, behaiour and body reactions of patients faced with diagnosis of cancer. JBUON.

[5] Mitchell A.J., Chan M., Bhatti H., Halton M., Grassi L., Johansen C., Meader N. (2011). Prevalence of depression, anxiety, and adjustment disorder in oncological, haematological, and palliative-care settings: A meta-analysis of 94 interview-based studies. Lancet Oncol..

[6] Klikovac T. (2019). Assesment of distress among oncology patients after the completion of the treatment. Topics.

[7] Muzzatti B., Mella S., Bomben F., Flabian C., Gipponi K., Piccinin M., Busato S., Annunziata M.A. (2016). Intensity and prevalence of depressive states in cancer inpatients: A large sample descriptive study. Eur. J. Cancer Care.

[8] Vucic V., Radovanovic S., Radevic S., Savkovic Z., Mihailovic N., Bosković Matic T.B. (2021). Mental Health Assessment of Cancer Patients: Prevalence and Predictive Factors of Depression and Anxiety. Iran. J. Public Health.

[9] Massie M.J. (2004). Prevalence of Depression in Patients with Cancer. JNCI Monogr..

[10] Pitman A., Suleman S., Hyde N., Hodgkiss A. (2018). Depression and anxiety in patients with cancer. BMJ.

[11] Linden W., Vodermaier A., MacKenzie R., Greig D. (2012). Anxiety and depression after cancer diagnosis: Prevalence rates by cancer type, gender, and age. J. Affect. Disord..

[12] Smith H.R. (2015). Depression in cancer patients: Pathogenesis, implications and treatment (Review). Oncol. Lett..

[13] Krebber A.M., Buffart L.M., Kleijn G., Riepma I.C., Bree R., Leemans C.R., Becker A., Brug J., van Straten A., Cujipers P. (2013). Prevalence of depression in cancer patients: A meta-analysis of diagnostic interviews and self-report instruments. Psycho-Oncology.

[14] Guven D.C., Sahin T.K., Aktepe O.H., Yildirim H.C., Aksoy S., Kilickap S. (2020). Perspectives, Knowledge, and Fears of Cancer Patients About COVID-19. Front. Oncol..

[15] Liu Y., Lu H., Wang W., Liu Q., Zhu C. (2020). Clinical risk factors for mortality in patients with cancer and COVID-19: A systematic review and meta-analysis of recent observational studies. Expert Rev. Anticancer. Ther..

[16] Wang Y.-H., Li J.-Q., Shi J.-F., Que J.-Y., Liu J.-J., Lappin J., Leung J., Ravindran A.V., Chen W.-Q., Qiao Y.-L. (2019). Depression and anxiety in relation to cancer incidence and mortality: A systematic review and meta-analysis of cohort studies. Mol. Psychiatry.

[17] Kassianos A.P., Georgiou A., Kyprianidou M., Lamnisos D., Ļubenko J., Constantinidou A. (2021). Mental Health and Adherence to COVID-19 Protective Behaviors among Cancer Patients during the COVID-19 Pandemic: An International, Multinational Cross-Sectional Study. Cancers.

[18] Momenimovahed Z., Salehiniya H., Hadavandsiri F., Allahqoli L., Günther V., Alkatout I. (2021). Psychological Distress Among Cancer Patients During COVID-19 Pandemic in the World: A Systematic Review. Front. Psychol..

[19] Chen G., Wu Q., Jiang H., Zhang H., Peng J., Hu J., Chen M., Zhong Y., Xie C. (2020). Fear of disease progression and psychological stress in cancer patients under the outbreak of COVID -19. Psycho-Oncology.

[20] Chen X., Wang L., Liu L., Jiang M., Wang W., Zhou X., Shao J. (2021). Factors associated with psychological distress among patients with breast cancer during the COVID-19 pandemic: A cross-sectional study in Wuhan, China. Support. Care Cancer.

[21] Cui Q., Cai Z., Li J., Liu Z., Sun S., Chen C., Wang G. (2020). The Psychological Pressures of Breast Cancer Patients During the COVID-19 Outbreak in China—A Comparison with Frontline Female Nurses. Front. Psychiatry.

[22] Gallagher S., Bennett K.M., Rope L. (2020). Loneliness and depression in patients with cancer during COVID-19. J. Psychosoc. Oncol..

[23] Wang Y., Duan Z., Ma Z., Mao Y., Li X., Wilson A., Qin H., Ou J., Peng K., Zhou F. (2020). Epidemiology of mental health problems among patients with cancer during COVID-19 pandemic. Transl. Psychiatry.

[24] Miaskowski C., Paul S.M., Snowberg K., Abbott M., Borno H., Chang S., Chen L.M., Cohen B., Hammer M.J., Kenfield S.A. (2020). Stress and Symptom Burden in Oncology Patients During the COVID-19 Pandemic. J. Pain Symptom Manag..

[25] Büntzel J., Micke O., Klein M., Büntzel J., Walter S., Keinki C., Huebner J. (2021). Take care or “German Angst”? Lessons from cancer care during COVID-19 pandemic in spring 2020. J. Cancer Res. Clin. Oncol..

[26] Levy I., Sharf G., Norman S., Tadmor T. (2021). The impact of COVID-19 on patients with hematological malignancies: The mixed-method analysis of an Israeli national survey. Support. Care Cancer.

[27] Rodrigues-Oliveira L., Kauark-Fontes E., Alves C.G.B., Tonaki J.O., Gueiros L.A., Moutinho K., Marta G.N., Barros L.R.C., Santos-Silva A.R., Brandão T.B. (2021). COVID-19 impact on anxiety and depression in head and neck cancer patients: A cross-sectional study. Oral Dis..

[28] Wong L.P., Lai L.L., See M.H., Alias H., Panaee M., Ting C.Y., Tok P.S.K. (2021). Psychological distress among cancer survivors during implementation of a nationwide Movement Control Order over the COVID-19 pandemic. Support. Care Cancer.

[29] Swainston J., Chapman B., Grunfeld E.A., Derakshan N. (2020). COVID-19 Lockdown and Its Adverse Impact on Psychological Health in Breast Cancer. Front. Psychol..

[30] Bargon C., Batenburg M., Stam L., Molen D.M., Dam I., Leij F., Baas I., Ernst M., Maarse W., Vermulst N. (2020). The impact of the COVID-19 pandemic on quality of life, physical and psychosocial wellbeing in breast cancer patients—A prospective, multicenter cohort study. Eur. J. Cancer.

[31] Gultekin M., Ak S., Ayhan A., Strojna A., Pletnev A., Fagotti A., Perrone A.M., Erzeneoglu B.E., Temiz B.E., Lemley B. (2021). Perspectives, fears and expectations of patients with gynaecological cancers during the COVID-19 pandemic: A Pan-European study of the European Network of Gynaecological Cancer Advocacy Groups (ENGAGe). Cancer Med..

[32] Hamlish T., Papautsky E.L. (2022). Differences in Emotional Distress Among Black and White Breast Cancer Survivors During the COVID-19 Pandemic: A National Survey. J. Racial Ethn. Health Disparities.

[33] Nardone V., Reginelli A., Vinciguerra C., Correale P., Calvanese M.G., Falivene S., Sangiovanni A., Grassi R., Di Biase A., Polifrone M.A. (2021). Mood Disorder in Cancer Patients Undergoing Radiotherapy During the COVID-19 Outbreak. Front. Psychol..

[34] Yildirim O.A., Poyraz K., Erdur E. (2021). Depression and anxiety in cancer patients before and during the SARS-CoV-2 pandemic: Association with treatment delays. Qual. Life Res..

[35] Klikovac T. (2022). The development of psycho-oncology worlwide and in Serbia—First steps and future plans. Topics.

[36] Ernst M., Beutel M.E., Brähler E. (2022). Cancer as a risk factor for distress and its interactions with sociodemographic variables in the context of the first wave of the COVID-19 pandemic in Germany. Sci. Rep..

[37] McAlonan G.M., Lee A.M., Cheung V., Cheung C., Tsang K.W., Sham P.C., Chua S.E., Wong J.G. (2007). Immediate and Sustained Psychological Impact of an Emerging Infectious Disease Outbreak on Health Care Workers. Can. J. Psychiatry.

[38] Wang C., Pan R., Wan X., Tan Y., Xu L., Ho C.S., Ho R.C. (2020). Immediate Psychological Responses and Associated Factors during the Initial Stage of the 2019 Coronavirus Disease (COVID-19) Epidemic among the General Population in China. Int. J. Environ. Res. Public Health.

[39] Mazza C., Ricci E., Biondi S., Colasanti M., Ferracuti S., Napoli C., Roma P. (2020). A Nationwide Survey of Psychological Distress among Italian People during the COVID-19 Pandemic: Immediate Psychological Responses and Associated Factors. Int. J. Environ. Res. Public Health.

[40] Jovanovic V., Gavrilov-Jerkovic V., Zuljevic D., Brdaric D. (2014). Psihometrijska evaluacija skale depresivnosti, anksioznosti i stresa–21 (DASS–21) na uzorku studenata u Srbiji [Psychometric Evaluation of Depression, Anxiety and Stress-21 Scale (DASS-21) on a Student Sample in Serbia]. Psihologija.

[41] Lovibond P.F. (1995). Manual for the Depression Anxiety Stress Scales.

[42] Vujčić I., Safiye T., Milikić B., Popović E., Dubljanin D., Dubljanin E., Dubljanin J., Čabarkapa M. (2021). Coronavirus Disease 2019 (COVID-19) Epidemic and Mental Health Status in the General Adult Population of Serbia: A Cross-Sectional Study. Int. J. Environ. Res. Public Health.

[43] Traunmüller C., Stefitz R., Gaisbachgrabner K., Schwerdtfeger A. (2020). Psychological correlates of COVID-19 pandemic in the Austrian population. BMC Public Health.

[44] Alkhamees A.A., Alrashed S.A., Alzunaydi A.A., Almohimeed A.S., Aljohani M.S. (2020). The psychological impact of COVID-19 pandemic on the general population of Saudi Arabia. Compr. Psychiatry.

[45] Juanjuan L., Santa-Maria C.A., Hongfang F., Lingcheng W., Pengcheng Z., Yuanbing X., Yuyan T., Zhongchun L., Bo D., Meng L. (2020). Patient-reported Outcomes of Patients with Breast Cancer During the COVID-19 Outbreak in the Epicenter of China: A Cross-sectional Survey Study. Clin. Breast Cancer.

[46] Arrieta O., Lara-Mejía L., Bautista-González E., Heredia D., Turcott J.G., Barrón F., Ramos-Ramírez M., Cabrera-Miranda L., Padilla M.S., Aguerrebere M. (2021). Clinical Impact of the COVID-19 Pandemic in Mexican Patients with Thoracic Malignancies. Oncologist.

[47] Roh S., Burnette C.E., Lee Y.-S., Giger J.T., Goins R.T., Petereit D.G., Lawler M.J., Lee K.H. (2019). Identifying risk and protective factors related to depressive symptoms among Northern Plains American Indian women cancer survivors. Women Health.

